# Neurodevelopmental Outcomes Among Offspring Exposed to Corticosteroid and B2-Adrenergic Agonists In Utero

**DOI:** 10.1001/jamanetworkopen.2023.39347

**Published:** 2023-10-24

**Authors:** Abir Nagata, Toshio Masumoto, Hidekazu Nishigori, Takatoshi Nakagawa, Shinji Otani, Youichi Kurozawa, and the Japan Environment and Children’s Study Group

**Affiliations:** 1 Department of Regenerative Dermatology, Graduate School of Medicine, Osaka University, Osaka, Japan; 2 Graduate School of Public Health, St Luke’s International University, Tokyo, Japan; 3 Division of Health Administration and Promotion, Faculty of Medicine, Tottori University, Tottori, Japan; 4 Department of Development and Environmental Medicine, Fukushima Medical Center for Children and Women, Fukushima Medical University Graduate School of Medicine, Fukushima, Japan; 5 International Platform for Dryland Research and Education, Tottori University, Tottori, Japan

## Abstract

**Importance:**

Corticosteroids and β2-adrenergic agonists are commonly used during pregnancy to treat asthma. However, offspring neurodevelopmental outcomes following in utero exposure to these medications remain unclear.

**Objective:**

To investigate the association between timing of in utero exposure to corticosteroids and β2-adrenergic agonists and offspring neurodevelopmental milestones during the first 3 years of life.

**Design, Setting, and Participants:**

This cohort study obtained data from the Japan Environment and Children’s Study, an ongoing birth cohort study conducted in collaboration with 15 Regional Centers across Japan. Participants were mother-offspring pairs who were recruited between January 1, 2011, and March 31, 2014. Data were analyzed between January and February 2023.

**Exposure:**

Corticosteroids and β2-adrenergic agonists were the exposure of interest. Timing of corticosteroid and β2-adrenergic agonist exposure included early pregnancy (weeks 0-12), mid- to late pregnancy (weeks >12), and both stages of pregnancy.

**Main Outcomes and Measures:**

Offspring neurodevelopmental milestones (communication, gross motor, fine motor, problem-solving, and personal-social skills) were assessed using the Japanese version of the Ages and Stages Questionnaires, 3rd edition, at 6, 12, 18, 24, 30, and 36 months.

**Results:**

In total, 91 460 mother-offspring pairs were analyzed. Among mothers, the mean (SD) age at delivery was 31.20 (5.05) years. Among offspring, 46 596 (50.9%) were males and 44 864 (49.1%) were females, of whom 66.4% had a gestational age of 39 to 41 weeks. During early, mid- to late, and both stages of pregnancy, 401 (0.4%), 935 (1.0%), and 568 (0.6%) offspring, respectively, were exposed to corticosteroids, whereas 170 (0.2%), 394 (0.4%), and 184 (0.2%), respectively, were exposed to β2-adrenergic agonists. No association of corticosteroid exposure during early, mid- to late, and both stages of pregnancy with all 5 neurodevelopmental milestones was found. Similarly, no association between β2-adrenergic agonist use during early pregnancy and all 5 neurodevelopmental milestones was observed. An association was found between β2-adrenergic agonist exposure during mid- to late pregnancy and delayed personal-social skills (adjusted odds ratio, 1.48; 95% CI, 1.01-2.32; *P* = .045).

**Conclusions and Relevance:**

Results of this study found no association between in utero corticosteroid and β2-adrenergic agonist exposure and offspring neurodevelopmental outcomes, regardless of the timing of exposure. Despite the limitations and low power of the study, the findings suggest that corticosteroids and β2-adrenergic agonists are safe for pregnant individuals with asthma and the neurodevelopment of their offspring.

## Key Points

**Question:**

Is the timing of in utero corticosteroid and β2-adrenergic agonist exposure associated with offspring neurodevelopmental outcomes during the first 3 years of life?

**Findings:**

In this cohort study of 91 460 mother-offspring pairs, no associations were found between corticosteroid and β2-adrenergic agonist exposure in early pregnancy, mid- to late pregnancy, or both stages of pregnancy and the development of communication, gross motor, fine motor, problem-solving, and personal-social skills in offspring, when compared with nonexposed offspring, during the first 3 years of life.

**Meaning:**

Findings of this study suggest that corticosteroids and β2-adrenergic agonists are safe for pregnant individuals with asthma and their offspring’s neurodevelopment.

## Teaser

This cohort study examines the parent-assessed communication, gross motor, fine motor, problem-solving, and personal-social skills in children whose mothers used corticosteroids and β2-adrenergic agonists for asthma during pregnancy.

## Introduction

Asthma is one of the foremost obstructive pulmonary diseases encountered during pregnancy, affecting 4% to 13% of females, and may require medicinal intervention to lessen complications.^1,2,3,4^ Complications of asthma exacerbation during pregnancy are associated with an increased risk of adverse maternal and fetal outcomes, including preterm delivery, low birth weight, congenital malformation, preeclampsia, and perinatal mortality, which affect the well-being of both the mother and offspring.^4,5,6^ Accordingly, optimizing and sustaining adequate asthma control during pregnancy with appropriate medications are recommended.^1,4^

Several medications are considered to be acceptable for treating asthma during pregnancy; however, some may have teratogenic potential and, hence, pose a risk to the fetus. During pregnancy, commonly used therapeutics include corticosteroids for their anti-inflammatory properties and β2-adrenergic agonists as bronchodilators.^7,8,9^ Evidence from animal studies suggests that these medications have the capability to permeate both the placenta and blood-brain barrier, leading to concerns regarding their biological potential to pose a risk to fetal neurodevelopment.^10,11,12,13^ However, evidence is lacking on offspring neurodevelopmental outcomes following in utero exposure to corticosteroids and β2-adrenergic agonists.

Existing information on cognitive and psychomotor developmental outcomes associated with maternal antiasthmatic medication use during pregnancy is inconsistent. For instance, some observational studies have found an association between in utero antiasthmatic medication exposure and subsequent neurophysiological, behavioral, and developmental disorders in offspring^14,15,16^; however, other studies have found that offspring neurodevelopmental outcomes are less likely to be associated with fetal antiasthmatic medication exposure.^17,18^ Currently, there exists a gap in the body of literature regarding the safety of asthma medications when used during pregnancy.^19^ Moreover, understanding the susceptibility to antiasthmatic medication exposure and its association with early-life offspring neurodevelopment is crucial for guiding clinical decisions. Thus, we aimed to investigate the association between timing (early, mid- to late, and both stages of pregnancy) of in utero exposure to corticosteroids and β2-adrenergic agonists and offspring neurodevelopmental milestones (communication, gross motor, fine motor, problem-solving, and personal-social skills) compared with nonexposed offspring during the first 3 years of life.

## Methods

Data for this cohort study were derived from the Japan Environment and Children’s Study (JECS), an ongoing prospective birth cohort study in Japan. The Ministry of the Environment’s Institutional Review Board on Epidemiological Studies and the ethics committees of all participating institutions reviewed and approved the JECS protocol. The JECS was conducted in accordance with the Declaration of Helsinki^20^ and the Ethical Guidelines for Medical and Health Research Involving Human Subjects, which were established by the Ministry of Education, Culture, Sports, Science and Technology and the Ministry of Health, Labour, and Welfare of Japan. All JECS participants provided written informed consent after the study aims and protocol were explained. The JECS approval and participants' consent also apply to the current study. We followed the Strengthening the Reporting of Observational Studies in Epidemiology (STROBE) reporting guideline.

### Study Design, Data Sources, and Participants

Details of the JECS design have been previously described.^21^ Briefly, the JECS began in 2011 to evaluate the role of environmental factors in the health and development of children in Japan.^21,22^ The project was designed to observe offspring prenatally until age 13 years and to operate in collaboration with 15 Regional Centers across Japan; more information on the JECS is provided in the eMethods in Supplement 1. Eligibility criteria were pregnancy and residency within the study area between January 1, 2011, and March 31, 2014. Among 104 062 fetal records from 103 060 pregnancies, 100 303 live births were included; thereafter, multiple births, preterm and postterm births, births with missing data on maternal antiasthmatic medication use, and births with missing or indeterminant offspring sex were excluded. The data used in this study were obtained from the JECS data set that was released in October 2019.

### Exposure

Corticosteroids (Anatomical Therapeutic Chemical code R01AD) and β2-adrenergic agonists (code R03AC or R03CC) were the exposure of interest in this study. Data on exposure variables were collected using 2 face-to-face prenatal interviews, based on a medication list, conducted by the research coordinator at each Regional Center. The interview questions included “Did you take medicine, drugs, or supplements in the past year?” Those participants who responded affirmatively were then asked about the timing of their medication use and the specific type of medication. In the first interview, which was conducted at the time of enrollment (during early to mid-pregnancy), participants were asked, “Did you take any corticosteroids (whether orally, injected, or inhaled) for the purpose of preventing asthma and/or β2-adrenergic agonists (orally or inhaled) 1 year prior [to] confirmation of pregnancy, between the confirmation of pregnancy and gestational age of 12 weeks, and after gestational age of 12 weeks until now?” In the second interview, which took place during mid- to late pregnancy, similar questions were repeated, with the exception of prepregnancy exposure. An affirmative response in interview 1 and interview 2 was considered as an exposure variable.

The timing of exposure to antiasthmatic medications was categorized into early (weeks 0-12), mid- to late (weeks >12), or both stages of pregnancy. The comparison group comprised offspring whose mothers did not use antiasthmatic medications during pregnancy. We also stratified the timing of exposure as follows: during early pregnancy (yes or no) and during mid- to late pregnancy (yes or no). Additionally, we assessed 1-year prepregnancy exposure and incorporated it as a negative control group, as prepregnancy exposure to antiasthmatic medications presumably is not a direct factor in offspring development; accordingly, a null result was anticipated.

### Developmental Assessment

Offspring neurodevelopmental outcomes between 6 and 36 months of age were assessed using the Japanese version of the Ages and Stages Questionnaires, 3rd edition (J-ASQ-3),^23^ which was sent by mail every 6 months post partum. The ASQ-3 is an age-specific screening tool completed by parents or primary guardians that measures developmental delays in 5 domains or milestones: communication, gross motor, fine motor, problem-solving, and personal-social skills^24^ (eMethods in Supplement 1). Achievement of neurodevelopmental milestones was assessed using 6 questions with the following answer options: yes (score: 10), sometimes (score: 5), or not yet (score: 0). Individual item scores were summed for a total score in each domain (range: 0-60). If 1 or 2 out of the 6 questions were left unanswered, the total score was multiplied by 1.2 (with 1 unanswered question) or 1.5 (with 2 unanswered questions), based on a score range of 0 to 60. Within the domain of gross motor skills assessment for offspring aged 2 years, 1 question was about behaviors that might have been exhibited previously but ceased due to acquisition of more advanced skills. When parents or guardians indicated not yet or sometimes for the simpler item and yes for the more advanced item, the response for the earlier item was revised to yes.

Child development was defined as a total score in each domain that was less than 2 SDs compared with the mean in the reference indicated as typical or potentially delayed (ie, requiring referral for further assessment of the evaluated domain) and was validated by Mezawa et al.^23^ The age-specific cutoff points of the J-ASQ-3 are provided in eTable 1 in Supplement 1. A previous study demonstrated that this threshold exhibited moderate sensitivity and specificity in identifying delays, ranging from any delay to severe delay, motor delay, and cognitive delay,^25^ and the threshold has been widely adopted in the assessment of Japanese children.^26,27^ In sensitivity analyses, we also used the present sample mean of less than 2 SDs as cutoff values for each J-ASQ-3 domain.

### Potential Confounders

To delineate assumptions regarding the causal pathway between antiasthmatic medication exposure and offspring neurodevelopment, we identified potential confounding variables through the literature^14,15,16,17,26,27^ and conceptualized it using a directed acyclic graph (eFigure 1 in Supplement 1) that included maternal age at delivery, marital status, educational level, history of prepregnancy asthma, and alcohol consumption during pregnancy; maternal and paternal smoking during pregnancy; household annual income; and offspring sex, which was hypothesized to be associated with exposure and outcome variables but not on the causal pathway. Additional factors were included in the sensitivity analysis: maternal prepregnancy body mass index (BMI), infertility treatment, psychological distress during pregnancy, gestational diabetes, and comedications (antibiotics, iron, and folic acid) and offspring gestational age, birth weight, breastfeeding duration, and nursery attendance. Covariate sources are provided in the eMethods in Supplement 1.

### Statistical Analysis

Descriptive statistics were used to outline the general characteristics of participants and offspring neurodevelopmental status. We used generalized estimating equations (GEEs) with robust SEs and an independent working correlation matrix structure to evaluate the association between the timing of corticosteroids and β2-adrenergic agonist exposure and repeatedly measured neurodevelopmental milestones using J-ASQ-3 (every 6 months, between age 6 and 36 months). We constructed GEE models with a binominal probability distribution and logit link function. Specifically, logistic regression analyses were conducted under GEEs, with dichotomized J-ASQ-3 domain scores as the outcome. These analyses were stratified by the timing of exposure (ie, no exposure during pregnancy vs early, mid- to late, or both stages of pregnancy), while being adjusted for confounding factors. Furthermore, we compared neurodevelopmental outcomes after early pregnancy exposure vs no exposure and after mid- to late pregnancy exposure vs no exposure.

Several sensitivity analyses were conducted to ensure the robustness of the main analysis. First, we created an additional model with further adjustment of maternal prepregnancy BMI, infertility treatment, psychological distress during pregnancy, gestational diabetes, and comedications (antibiotics, iron, and folic acid) and offspring gestational age, birth weight, breastfeeding duration, and nursery attendance. Second, to account for incomplete data, we imputed missing data using multiple imputation techniques after considering data that were missing at random. We imputed 10 data sets using fully conditional specification for each variable and combined the estimates in GEEs. Third, we applied the present sample mean of less than (2 × SD) as cutoff values for each J-ASQ-3 domain. Fourth, we applied linear multivariable GEE models using the continuous J-ASQ-3 domain score as the outcome.

Subgroup analyses were performed to understand the role of offspring sex and maternal history of prepregnancy asthma. We assessed the effect modification using the interaction term in the GEE models. A 2-tailed *P* < .05 indicated statistical significance. For subgroup analysis, *P* < .005 was considered to avoid type I error. The statistical analysis was performed between January and February 2023 using IBM SPSS Statistics for Windows/Macintosh, version 25.0 (IBM Corp).

## Results

### Cohort Characteristics

The final sample comprised 91 460 mother-offspring pairs (Figure). Table 1 shows the participants’ characteristics. Among 91 460 mothers, the mean (SD) age at delivery was 31.20 (5.05) years. Additionally, 4.5% of mothers smoked and 2.8% consumed alcohol during pregnancy, while 10.9% had a history of prepregnancy asthma. Among 91 460 offspring, 46 596 (50.9%) were males and 44 864 (49.1%) were females, of whom 66.4% had a gestational age of 39 to 41 weeks. More than 65% of offspring were breastfed during the first 12 months of life. During early, mid- to late, and both stages of pregnancy, 0.4%, 1.0%, and 0.6% of offspring, respectively, were exposed to corticosteroids, whereas 0.2%, 0.4%, and 0.2%, respectively, were exposed to β2-adrenergic agonists. Table 2 and eFigure 2 in Supplement 1 summarize the neurodevelopmental milestones of the offspring and the J-ASQ-3 domain scores according to age.

**Figure.**
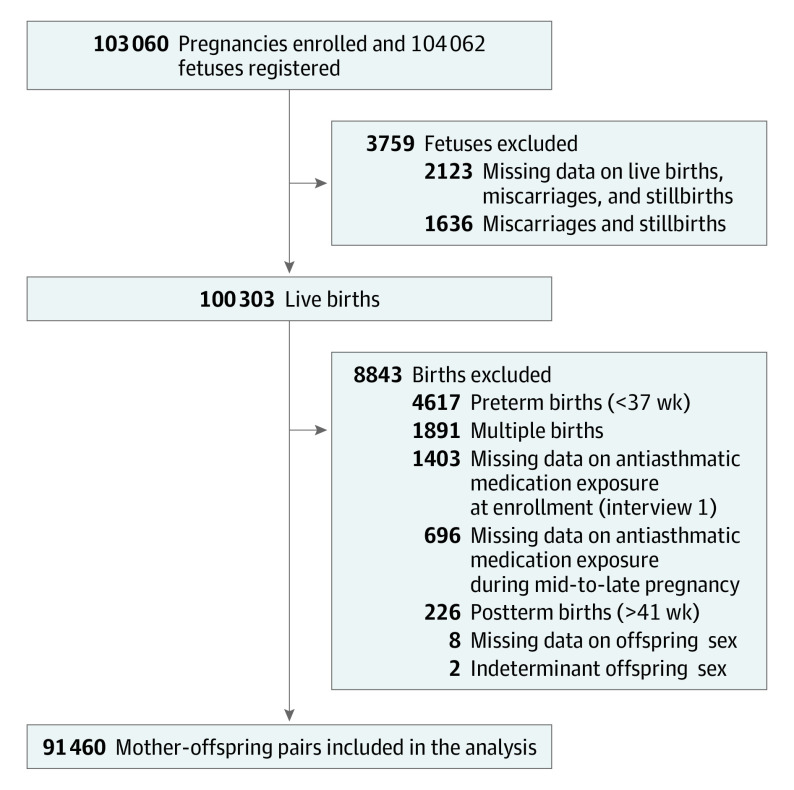
Flowchart of Participants

**Table 1.** Distribution of Participant Characteristics by Corticosteroid and/or β2-Adrenergic Agonist Exposure

Characteristic	Participants, No. (%)		
	Total (n = 91 460)	Unexposed (n = 89 138)	Exposed (n = 2322)
**Maternal**			
Age at delivery, y			
<25	7218 (7.9)	7112 (10.1)	106 (6.5)
25-29	19 845 (21.7)	19 446 (27.6)	399 (24.3)
30-34	25 519 (27.9)	24 917 (35.3)	602 (36.7)
≥35	19 556 (21.4)	19 023 (27.0)	533 (32.5)
Missing data	19 322 (21.1)	NA	NA
Marital status			
Single	3181 (3.5)	3114 (3.5)	67 (2.9)
Married	86 849 (95.0)	84 636 (95.7)	2213 (96.1)
Divorced or widowed	739 (0.8)	717 (0.8)	22 (1.0)
Missing data	691 (0.7)	NA	NA
Educational level			
≤High school diploma	32 902 (36.0)	32 115 (36.4)	787 (34.2)
College attendance	38 081 (41.6)	37 049 (42.0)	1032 (44.8)
≥Bachelor’s degree	19 637 (21.5)	19 153 (21.6)	484 (21.0)
Missing data	840 (0.9)	NA	NA
Prepregnancy BMI			
<18.5	14 917 (16.3)	14 571 (16.8)	346 (15.3)
18.5-24.9	65 680 (71.8)	64 077 (73.6)	1603 (70.8)
≥25	8650 (9.5)	8336 (9.6)	314 (13.9)
Missing data	2213 (2.4)	NA	NA
Smoking during pregnancy			
No	86 274 (94.3)	84 092 (95.5)	2182 (95.2)
Yes	4100 (4.5)	3990 (4.5)	110 (4.8)
Missing data	1086 (1.2)	NA	NA
Alcohol consumption during pregnancy			
No	87 814 (96.0)	85 594 (97.2)	2220 (97.0)
Yes	2562 (2.8)	2494 (2.8)	68 (3.0)
Missing data	1084 (1.2)	NA	NA
Infertility treatment			
No	85 376 (93.3)	83 204 (93.7)	2172 (93.9)
Yes	5712 (6.2)	5571 (6.3)	141 (6.1)
Missing data	372 (0.5)	NA	NA
Psychological distress during pregnancy, score^a^			
0-4	61 847 (67.6)	60 360 (68.2)	1487 (64.4)
≥5	28 950 (31.7)	28 127 (31.8)	823 (35.6)
Missing data	663 (0.7)	NA	NA
Gestational diabetes			
No	88 808 (97.1)	86 558 (97.4)	2250 (97.2)
Yes	2421 (2.6)	2356 (2.6)	65 (2.8)
Missing data	231 (0.3)	NA	NA
History of prepregnancy asthma			
No	81 200 (88.8)	80 006 (90.1)	1194 (51.6)
Yes	9959 (10.9)	8837 (9.9)	1122 (48.4)
Missing data	301 (0.3)	NA	NA
Comedication: antibiotics			
No	71 692 (78.4)	69 990 (78.7)	1 702 (73.5)
Yes	19 537 (21.3)	18 924 (21.3)	613 (26.5)
Missing data	231 (0.3)		
Iron			
No	52 415 (57.3)	50 999 (57.4)	1416 (61.2)
Yes	38 814 (42.4)	37 915 (42.6)	899 (38.8)
Missing data	231 (0.3)	NA	NA
Folic acid			
No	89 354 (97.7)	87 089 (97.9)	2 265 (97.8)
Yes	1875 (2.0)	1825 (2.1)	50 (2.2)
Missing data	231 (0.3)	NA	NA
**Household**			
Paternal smoking			
No	47 682 (52.1)	46 427 (53.3)	1255 (55.4)
Yes	41 766 (45.7)	40 756 (46.7)	1010 (44.6)
Missing data	2012 (2.2)	NA	NA
Annual income, in millions, ¥^b^			
<4	34 058 (37.2)	33 182 (40.2)	876 (40.4)
4-8	41 499 (45.4)	40 438 (49.0)	1061 (48.9)
≥8	9109 (10.0)	8876 (10.8)	233 (10.7)
Missing data	6 794 (7.4)	NA	NA
**Offspring**			
Sex			
Male	46 596 (50.9)	45 403 (50.9)	1193 (51.4)
Female	44 864 (49.1)	43 735 (49.1)	1129 (48.6)
Missing data	NA	NA	NA
Gestational age, wk			
37-38	30 470 (33.3)	29 601 (33.3)	869 (37.5)
39-41	60 759 (66.4)	59 313 (66.7)	1446 (62.5)
Missing data	231 (0.3)	NA	NA
Birth weight, g			
<2500	4953 (5.4)	4821 (5.4)	132 (5.7)
2500-4000	85 415 (93.4)	83 254 (93.7)	2161 (93.4)
>4000	810 (0.9)	790 (0.9)	20 (0.9)
Missing data	282 (0.3)	NA	NA
Breastfeeding duration			
1-6 mo			
No	5544 (6.1)	5403 (6.4)	141 (6.4)
Yes	80 497 (88.0)	78 450 (93.6)	2047 (93.6)
Missing data	5419 (5.9)	NA	NA
7-12 mo			
No	21 398 (23.4)	20 781 (25.6)	617 (28.5)
Yes	61 946 (67.7)	60 398 (74.4)	1548 (71.5)
Missing data	8116 (8.9)	NA	NA
Nursery attendance^c^			
No	60 637 (66.3)	59 097 (73.1)	1540 (71.6)
Yes	22 350 (24.4)	21 738 (26.9)	612 (28.4)
Missing data	8473 (9.3)	NA	NA

Abbreviations: BMI, body mass index (calculated as weight in kilograms divided by height in meters squared); K6, Kessler Psychological Distress Scale (range: 0 to ≥5, with the highest score indicating poor mental health); NA, not applicable.

^a^ Maternal psychological distress during pregnancy was measured by the K6 scale.

^b^ To convert yen to US dollars, multiply by 0.00677.

^c^ Going to nursery school at 1 year of age.

**Table 2.** Offspring Neurodevelopmental Milestones (n = 91 460)^a^

Offspring age, mo	Developmental status	J-ASQ-3 domains, No. (%)				
		Communication skills	Gross motor skills	Fine motor skills	Problem-solving skills	Personal-social skills
6	Typical	79 219 (99.3)	71 504 (89.7)	75 487 (94.9)	71 118 (89.2)	76 676 (96.3)
	Delayed	522 (0.7)	8226 (10.3)	4025 (5.1)	8616 (10.8)	2951 (3.7)
12	Typical	75 662 (99.9)	71 412 (94.3)	71 288 (94.1)	71 668 (94.7)	74 594 (98.8)
	Delayed	100 (0.1)	4354 (5.7)	4439 (5.9)	3990 (5.3)	938 (1.2)
18	Typical	69 795 (97.8)	68 055 (95.3)	68 211 (95.6)	68 060 (95.9)	71 905 (98.5)
	Delayed	1598 (2.2)	3357 (4.7)	3160 (4.4)	2909 (4.1)	1110 (1.5)
24	Typical	70 274 (96.1)	68 906 (94.2)	71 461 (97.8)	69 858 (95.8)	70 955 (97.2)
	Delayed	2847 (3.9)	4216 (5.8)	1594 (2.2)	3062 (4.2)	2060 (2.8)
30	Typical	67 922 (95.2)	68 315 (95.7)	66 938 (94.2)	67169 (94.3)	68 897 (96.7)
	Delayed	3443 (4.8)	3078 (4.3)	4109 (5.8)	4042 (5.7)	2371 (3.3)
36	Typical	70 195 (96.1)	69 939 (95.6)	67 452 (92.6)	67 212 (92.8)	70 615 (96.8)
	Delayed	2837 (3.9)	3193 (4.4)	5403 (7.4)	5238 (7.2)	2345 (3.2)

Abbreviation: J-ASQ-3, Japanese version of the Ages and Stages Questionnaires, 3rd edition.

^a^ Child development was a total score in each domain that was less than 2 SDs compared with the mean in the reference indicated as typical or potentially delayed developmental status. The cutoff values of J-ASQ-3 are provided in eTable 1 in Supplement 1.

### Association of Timing of In Utero Corticosteroid and β2-Adrenergic Agonist Exposure With Offspring Neurodevelopment Outcomes 

Table 3 reports the association of the timing of in utero corticosteroid and β2-adrenergic agonist exposure with offspring neurodevelopment as estimated with GEE models. We found no association of corticosteroid exposure during early, mid- to late, and both stages of pregnancy with all 5 neurodevelopmental milestones. Similarly, no association between β2-adrenergic agonist use during early pregnancy and all 5 neurodevelopmental milestones was observed. The odds of having delayed communication (adjusted odds ratio [AOR], 1.30; 95% CI, 0.79-2.15), gross motor (AOR, 1.27; 95% CI, 0.90-1.78), fine motor (AOR, 1.21; 95% CI, 0.83-1.75), and problem-solving (AOR, 1.16; 95% CI, 0.83-1.62) skills were higher in offspring with β2-adrenergic agonist exposure during mid- to late pregnancy compared with nonexposed offspring; however, these findings were not statistically significant. We observed an association between β2-adrenergic agonist exposure during mid- to late pregnancy and delayed personal-social skills (AOR, 1.48; 95% CI, 1.01-2.32; *P* = .045). Nevertheless, no other neurodevelopmental milestones were associated with other timing of β2-adrenergic agonist exposure (Table 3), including prepregnancy (eTable 2 in Supplement 1). Furthermore, we found no association with neurodevelopmental delays when we compared early pregnancy exposure vs no exposure with mid- to late pregnancy exposure vs no exposure (Table 4).

**Table 3.** Association of Corticosteroids and β2-Adrenergic Agonists Exposure In Utero With Offspring Neurodevelopmental Milestones

	Offspring, No. (%)	J-ASQ-3 domains, AOR (95% CI)^a^				
		Communication skills	Gross motor skills	Fine motor skills	Problem-solving skills	Personal-social skills
**Corticosteroids**						
Unexposed, during pregnancy	87 677 (98.0)	1 [Reference]	1 [Reference]	1 [Reference]	1 [Reference]	1 [Reference]
Exposed, early pregnancy	401 (0.4)	1.00 (0.59-1.67)	0.83 (0.59-1.16)	0.89 (0.66-1.22)	0.96 (0.71-1.29)	0.93 (0.57-1.52)
Exposed, mid- to late pregnancy	935 (1.0)	1.09 (0.78-1.52)	0.97 (0.78-1.20)	0.93 (0.75-1.16)	0.95 (0.77-1.18)	0.99 (0.72-1.37)
Exposed, both early and mid- to late pregnancy	568 (0.6)	1.19 (0.80-1.77)	1.08 (0.82-1.42)	1.18 (0.92-1.50)	1.15 (0.91-1.46)	0.89 (0.56-1.41)
**β2-adrenergic agonists**						
Unexposed, during pregnancy	90 278 (99.2)	1 [Reference]	1 [Reference]	1 [Reference]	1 [Reference]	1 [Reference]
Exposed, early pregnancy	170 (0.2)	0.71 (0.23-2.21)	0.99 (0.56-1.74)	0.67 (0.38-1.17)	0.81 (0.49-1.35)	0.68 (0.24-1.95)
Exposed, mid- to late pregnancy	394 (0.4)	1.30 (0.79-2.15)	1.27 (0.90-1.78)	1.21 (0.83-1.75)	1.16 (0.83-1.62)	1.48 (1.01-2.32)^b^
Exposed, both early and mid- to late pregnancy	184 (0.2)	1.33 (0.68-2.62)	0.79 (0.51-1.21)	0.81 (0.51-1.28)	1.05 (0.69-1.60)	0.52 (0.25-1.07)

Abbreviations: AOR, adjusted odds ratio; J-ASQ-3, Japanese version of the Ages and Stages Questionnaires, 3rd edition.

^a^ Adjusted for maternal age at delivery, marital status, educational level, history of prepregnancy asthma, and alcohol consumption during pregnancy; maternal and paternal smoking during pregnancy; household annual income; and offspring sex.

^b^ *P* < .05.

**Table 4.** Association of Corticosteroids and β2-Adrenergic Agonist Exposure During Early and Mid- to Late Pregnancy With Offspring Neurodevelopmental Milestones

	Offspring, No. (%)	J-ASQ-3 domains, AOR (95% CI)^a^				
		Communication skills	Gross motor skills	Fine motor skills	Problem-solving skills	Personal-social skills
**Corticosteroids**						
Exposed, early pregnancy						
No	90 491 (98.9)	1 [Reference]	1 [Reference]	1 [Reference]	1 [Reference]	1 [Reference]
Yes	969 (1.1)	1.00 (0.72-1.38)	0.93 (0.75-1.16)	0.99 (0.81-1.20)	0.99 (0.82-1.21)	0.82 (0.58-1.16)
Exposed, mid- to late pregnancy						
No	89 957 (98.4)	1 [Reference]	1 [Reference]	1 [Reference]	1 [Reference]	1 [Reference]
Yes	1503 (1.6)	1.05 (0.81-1.38)	0.97 (0.81-1.15)	0.99 (0.84-1.18)	0.99 (0.84-1.16)	0.85 (0.64-1.12)
**β2-adrenergic agonists**						
Exposed, early pregnancy						
No	91 106 (99.6)	1 [Reference]	1 [Reference]	1 [Reference]	1 [Reference]	1 [Reference]
Yes	354 (0.4)	0.96 (0.52-1.78)	0.92 (0.64-1.31)	0.74 (0.52-1.06)	0.93 (0.67-1.29)	0.61 (0.31-1.18)
Exposed, mid- to late pregnancy						
No	90 882 (99.4)	1 [Reference]	1 [Reference]	1 [Reference]	1 [Reference]	1 [Reference]
Yes	578 (0.6)	1.37 (0.91-2.06)	1.13 (0.85-1.51)	1.11 (0.82-1.52)	1.14 (0.87-1.50)	1.21 (0.80-1.83)

Abbreviations: AOR, adjusted odds ratio; J-ASQ-3, Japanese version of the Ages and Stages Questionnaires, 3rd edition.

^a^ Adjusted for maternal age at delivery, marital status, educational level, history of prepregnancy asthma, and alcohol consumption during pregnancy; maternal and paternal smoking during pregnancy; household annual income; and offspring sex.

### Sensitivity and Subgroup Analyses

Results of the sensitivity analyses on adjusted models were consistent with findings of the main analyses (eTable 3 in Supplement 1). In models with missing value imputation, models with a sample mean of less than (2 × SD) as a cutoff on the J-ASQ-3, and linear multivariate models, we found no association with any timing of corticosteroid and β2-adrenergic agonist exposure during pregnancy with any of the 5 neurodevelopmental milestones (eTables 4, 5, and 6 in Supplement 1). In the subgroup analysis, we observed an increased risk of delayed problem-solving skills (AOR, 1.65; 95% CI, 1.11-2.45; *P* for interaction =.006) in males compared with females following β2-adrenergic agonist use during mid- to late pregnancy; however, this finding was not statistically significant (eTable 7 in Supplement 1). Furthermore, no effect modification was observed between corticosteroid and β2-adrenergic agonist exposure and offspring neurodevelopment with maternal history of prepregnancy asthma (eTable 8 in Supplement 1).

## Discussion

In this cohort study, we found no association between any timing of corticosteroid exposure during pregnancy and communication, gross motor, fine motor, problem-solving, and personal-social skills. However, we observed an association between β2-adrenergic agonist exposure during mid- to late pregnancy and delayed personal-social skills. In contrast, no other factor affecting neurodevelopmental milestones was associated with β2-adrenergic agonist during the exposure window of interest compared with unexposed offspring, collectively suggesting that no reasonable neurodevelopmental outcomes were associated with in utero corticosteroid and β2-adrenergic agonist exposure.

The findings were consistent with those of recent studies^17,28^ that investigated susceptible antiasthmatic medication exposure windows of neurocognitive development. A case-control study showed no evidence of an association between β2-adrenergic agonists and autism spectrum disorder risk.^17^ In a population-based and family-based case-control study, no association was found between inhaled corticosteroids and β2-adrenergic agonist use during pregnancy and offspring autism spectrum disorder.^18^ Similarly, a cohort study of 961 202 children reported no increased risk of attention-deficit/hyperactivity disorder following in utero exposure to antiasthmatic medication.^28^ Liang et al^29^ suggested an increased risk of attention-deficit/hyperactivity disorder following in utero β2-adrenergic agonist exposure; however, they also acknowledged that the association may be confounded by indication. Still, these observations may not be comparable with each other or with the present study’s findings due to variations in study designs, sample sizes, and assessment tools for medication exposure and offspring developmental outcomes.

Generally, most females of reproductive age with asthma were concerned about the implications of pharmacotherapy for fetal delivery and development, exhibiting reluctance to use medication during pregnancy.^3,30^ Current asthma management primarily relies on inhalation therapy; in Japan, inhaled corticosteroids and inhaled β2-adrenergic agonists are recommended as the first-choice treatment for asthma during pregnancy.^30^ Drug intake by inhalation may be associated with lower systemic exposure and, accordingly, may be less likely to reach the fetus, resulting in a decreased risk of adverse effects.

Animal experiments demonstrate that administering antiasthmatic medications during prenatal or early postnatal periods disrupts brain development, leading to biochemical and architectural abnormalities and changes in neurotransmitter pathways in the immature brain.^31,32^ However, caution must be exercised when comparing animal and human studies, as β2-adrenergic agonists are mainly administered subcutaneously in animals, not by inhalation.

We noticed an association between β2-adrenergic agonist exposure during mid- to late pregnancy and personal-social skills; however, it can be argued that medication exposure during the second and third trimesters is less likely to be associated with teratogenesis. These associations could be due to underlying disorders, pathological conditions, or environmental factors. Conversely, the observation may be probable when reflecting on the dynamic neurodevelopmental process that encompasses the fetal period to early childhood, as neuroplasticity is a complex process that occurs during time-sensitive periods of prenatal and postnatal brain development during which the brain is most amenable to change.^33,34^

A potential confounder of the association between maternal antiasthmatic medication use and subsequent offspring development is the maternal history of asthma during pregnancy.^18,28^ We found no evidence of confounding by a maternal history of prepregnancy asthma, consistent with those of several studies.^35,36,37^ Furthermore, we examined the effect modification by sex; the results suggested no sex differences regarding the association between in utero antiasthmatic medication exposure and offspring development. Yet, further replication in high-powered studies across diverse contexts and long-term neurodevelopmental outcomes is warranted. Nevertheless, this study’s findings provide new insights for clinicians and pregnant individuals in asthma treatment during pregnancy. Moreover, to our knowledge, the current study is the largest in Japan to examine offspring neurodevelopmental outcomes after in utero corticosteroid and β2-adrenergic agonist exposure using a large birth cohort data set of populations from both rural and urban areas.^22,38^ Moreover, we included a wide range of confounding factors and longitudinal outcome data that few previous studies have considered.

### Limitations

This study has limitations. First, although the JECS has a substantial sample size and demographic similarities to the Japanese population, generalization to other populations should be performed with caution because the JECS does not adhere strictly to a population-based approach.^39^ Second, the JECS was not primarily designed to evaluate the association of antiasthmatic medications with offspring development. Therefore, there is a lack of information regarding maternal asthma severity during pregnancy, medication dose, duration, and route of administration as well as participants’ capacity to accurately report their medication use, including both medication types and timing, which may have confounded the true association. Third, a recall bias or exposure misclassification may have occurred, although we collected antiasthmatic medication exposure information by face-to-face interviews. Fourth, we had a limited number of exposed cases, specifically with β2-adrenergic agonists; thus, there was a low study power in some of the analyses. Fifth, observer bias may have affected child neurodevelopment data, despite using the J-ASQ-3. Sixth, although the analyses were adjusted for several confounders, it is likely that residual confounding through unmeasured covariates may have remained.

## Conclusions

In this cohort study, we observed no association between the timing of in utero corticosteroid and β2-adrenergic agonist exposure and offspring neurodevelopment milestones during the first 3 years of life. Despite the study’s limitations and its low power, these findings suggest that corticosteroids and β2-adrenergic agonists could be considered safe for use by pregnant individuals with asthma and safe for the neurodevelopment of their offspring. Additionally, the findings may inform choices regarding the management of maternal asthma during pregnancy.

## Notes

Supplement 1. **eMethods.** Supplementary Methods **eTable 1.** Validated Japanese Version of the Ages and Stages Questionnaires, Third Edition (J-ASQ-3), Cutoff Values **eTable 2.** Association of Pre-Pregnancy Exposure to Corticosteroids and β2-Adrenergic Agonists (β2AAs) With Offspring Neurodevelopmental Milestones **eTable 3.** Sensitivity Analysis on the Association of Timing of In-Utero Exposure to Corticosteroids and β2AAs With Offspring Neurodevelopmental Milestones in Additionally Adjusted Generalized Estimating Equation (GEE) Models **eTable 4.** Sensitivity Analysis on the Association of Timing of In-Utero Exposure to Corticosteroids and β2AAs With Offspring Neurodevelopmental Milestones in GEE Models With Multiple Imputations of Missing Values **eTable 5.** Sensitivity Analysis on the Association of Timing of In-Utero Exposure to Corticosteroids and β2AAs With Offspring Neurodevelopmental Milestones in GEE Models With Sample Mean Less Than (2× Standard Deviation [SD]) as Cutoff Values for Each J-ASQ-3 Domain **eTable 6.** Sensitivity Analysis on the Association of Timing of In-Utero Exposure to Corticosteroids and β2AAs With Offspring Neurodevelopmental Milestones in Linear Multivariable GEE Models **eTable 7.** Subgroup Analysis of the Association of Timing of In-Utero Exposure to Corticosteroids and β2AAs With Offspring Neurodevelopmental Milestones According to the Child’s Sex **eTable 8.** Subgroup Analysis of the Association of Timing of In-Utero Exposure to Corticosteroids and β2AAs With Offspring Neurodevelopmental Milestones According to the Maternal History of Pre-Pregnancy Asthma **eFigure 1.** Directed Acyclic Graph for the Association Between In-Utero Corticosteroids and β2AA Exposure and Offspring Neurodevelopment **eFigure 2.** Scores of J-ASQ-3 According to Age
eReference

Supplement 2.
Nonauthor Collaborators

Supplement 3.
Data Sharing Statement
