# Supplementary material for: Neurodevelopmental Outcomes Among Offspring Exposed to Corticosteroid and B2-Adrenergic Agonists In Utero
Source: JAMA Netw Open. 2023 Oct 24;6(10):e2339347. doi: 10.1001/jamanetworkopen.2023.39347 (PMC10599123; doi:10.1001/jamanetworkopen.2023.39347)
Supplement: Supplement 1. — eMethods. Supplementary Methods eTable 1. Validated Japanese Version of the Ages and Stages Questionnaires, Third Edition (J-ASQ-3), Cutoff Values eTable 2. Association of Pre-Pregnancy Exposure to Corticosteroids and β2-Adrenergic Agonists (β2AAs) With Offspring Neurodevelopmental Milestones eTable 3. Sensitivity Analysis on the Association of Timing of In-Utero Exposure to Corticosteroids and β2AAs With Offspring Neurodevelopmental Milestones in Additionally Adjusted Generalized Estimating Equation (GEE) Models eTable 4. Sensitivity Analysis on the Association of Timing of In-Utero Exposure to Corticosteroids and β2AAs With Offspring Neurodevelopmental Milestones in GEE Models With Multiple Imputations of Missing Values eTable 5. Sensitivity Analysis on the Association of Timing of In-Utero Exposure to Corticosteroids and β2AAs With Offspring Neurodevelopmental Milestones in GEE Models With Sample Mean Less Than (2× Standard Deviation [SD]) as Cutoff Values for Each J-ASQ-3 Domain eTable 6. Sensitivity Analysis on the Association of Timing of In-Utero Exposure to Corticosteroids and β2AAs With Offspring Neurodevelopmental Milestones in Linear Multivariable GEE Models eTable 7. Subgroup Analysis of the Association of Timing of In-Utero Exposure to Corticosteroids and β2AAs With Offspring Neurodevelopmental Milestones According to the Child’s Sex eTable 8. Subgroup Analysis of the Association of Timing of In-Utero Exposure to Corticosteroids and β2AAs With Offspring Neurodevelopmental Milestones According to the Maternal History of Pre-Pregnancy Asthma eFigure 1. Directed Acyclic Graph for the Association Between In-Utero Corticosteroids and β2AA Exposure and Offspring Neurodevelopment eFigure 2. Scores of J-ASQ-3 According to Age eReference [file jamanetwopen-e2339347-s001.pdf]

Supplemental Online Content

Nagata A, Masumoto T, Nishigori H, Nakagawa T, Otani S, Kurozawa Y; Japan Environment and Children's Study Group. Association of in utero corticosteroid and β2-adrenergic agonist exposure with offspring neurodevelopmental outcomes. *JAMA Netw Open*. 2023;6(10):e2339347. doi:10.1001/jamanetworkopen.2023.39347

eMethods. Supplementary Methods
eTable 1. Validated Japanese Version of the Ages and Stages Questionnaires, Third Edition (J-ASQ-3), Cutoff Values
eTable 2. Association of Pre-Pregnancy Exposure to Corticosteroids and β2-Adrenergic Agonists (β2AAs) With Offspring Neurodevelopmental Milestones
eTable 3. Sensitivity Analysis on the Association of Timing of In-Utero Exposure to Corticosteroids and β2AAs With Offspring Neurodevelopmental Milestones in Additionally Adjusted Generalized Estimating Equation (GEE) Models
eTable 4. Sensitivity Analysis on the Association of Timing of In-Utero Exposure to Corticosteroids and β2AAs With Offspring Neurodevelopmental Milestones in GEE Models With Multiple Imputations of Missing Values
eTable 5. Sensitivity Analysis on the Association of Timing of In-Utero Exposure to Corticosteroids and β2AAs With Offspring Neurodevelopmental Milestones in GEE Models With Sample Mean Less Than (2×Standard Deviation [SD]) as Cutoff Values for Each J-ASQ-3 Domain
eTable 6. Sensitivity Analysis on the Association of Timing of In-Utero Exposure to Corticosteroids and β2AAs With Offspring Neurodevelopmental Milestones in Linear Multivariable GEE Models
eTable 7. Subgroup Analysis of the Association of Timing of In-Utero Exposure to Corticosteroids and β2AAs With Offspring Neurodevelopmental Milestones According to the Child's Sex
eTable 8. Subgroup Analysis of the Association of Timing of In-Utero Exposure to Corticosteroids and β2AAs With Offspring Neurodevelopmental Milestones According to the Maternal History of Pre-Pregnancy Asthma
eFigure 1. Directed Acyclic Graph for the Association Between In-Utero Corticosteroids and β2AA Exposure and Offspring Neurodevelopment
eFigure 2. Scores of J-ASQ-3 According to Age
eReference

This supplemental material has been provided by the authors to give readers additional information about their work.

© 2023 Nagata A et al. *JAMA Network Open*

eMethods

JECS Implementation Framework

The Japan Environment and Children's Study (JECS) operates as a national project funded directly by the Ministry of Environment. It consists of three-tier centers: the National Center for JECS at the National Institute for Environmental Studies leads the initiative, while the National Center for Child Health and Development provides medical expertise as the Medical Support Center. This collaboration extends to 15 Regional Centers: Hokkaido, Miyagi, Fukushima, Chiba, Kanagawa, Koshin, Toyama, Aichi, Kyoto, Osaka, Hyogo, Tottori, Kochi, Fukuoka, and South Kyusyu/Okinawa Regional Centers, situated in universities. These Regional Centers manage participant recruitment and follow-up activities in their respective Study Areas, working in tandem with local governments.

Study Areas

The 15 Regional Centers were strategically chosen to ensure the applicability and generalizability of the JECS findings to the broader Japanese population. These centers encompass a wide range of geographical regions, featuring varying degrees of urbanization and land development, spanning from urban and suburban to rural settings.

The selection of Regional Centers followed a competitive process, inviting proposals from universities and research institutions. These proposals outlined coverage areas, population considerations, recruitment strategies, organizational structures, regional coordination, and available resources. Comprising one or more Study Areas each, the Regional Centers cater to populations of 130 000 to 600 000 residents. Assuming a 1% birth rate, each Regional Center anticipates an annual average of 4 400 births, ranging from 1 300 to 6 000.

The JECS aims to encompass 50% of the total births within the covered areas. To achieve this, the chosen Regional Centers are tasked with enlisting 3 000 to 9 000 pregnant women over a 3-year span, culminating in 100 000 participants across all 15 centers. To optimize participant engagement, Regional Centers have established JECS regional liaisons in collaboration with local governmental bodies and healthcare providers. The Study Areas are demarcated within administrative units such as municipalities, fostering robust cooperation with local governments. This arrangement facilitates the collection of crucial health statistics, including birth counts, sex ratios, birth weights, morbidities, and mortalities. Moreover, it enhances the potential for successful follow-up and participant retention.

Study Participants

The enrollment goal was 100 000 pregnant women. Recruitment commenced in January 2011 and spanned 3 years until March 2014. In Japan, there were 1 070 025 births in 2009. The targeted enrollment of 100 000 over 3 years (approximately 33 333 annually) accounts for approximately 3% of all Japanese newborns. While partners can participate, their involvement is not obligatory.

Participant eligibility criteria for expectant mothers are as follows:

1. Residency within the Study Areas during recruitment and an intention to remain in Japan in the foreseeable future.
2. Expected delivery date falling between August 1, 2011 and mid-2014.
3. Capability to engage in the study, including proficiency in Japanese and completion of self-administered questionnaires.

Individuals residing outside the Study Areas, even if they visited cooperating healthcare providers within those areas, were not included in the study.

Recruitment and Follow-up Procedures

The JECS employs comprehensive strategies to engage expecting mothers within study areas, targeting a recruitment rate exceeding 50% of eligible individuals. Two primary recruitment protocols are utilized:

1. Provider-mediated Community-based Recruitment: Expecting mothers are approached during their initial prenatal examination at collaborating healthcare providers, particularly obstetric facilities.
2. Mother-Child Health Handbook Recruitment: Recruitment also occurs at local government offices when issuing Mother-Child Health Handbooks, a standard booklet provided to pregnant women in Japan for accessing municipal pregnancy, delivery, and childcare services.

Participation consent is collected from individual mothers, partners, and parent/guardians of children. Participants retain the freedom to withdraw from the study at any point. Those who do not consent or cannot be contacted during pregnancy are excluded. Mothers planning to give birth at their parents' residence are ineligible, unless accessible to Regional Centers. While the sampling plan of the JECS is well-structured, recruitment is not entirely randomized due to engagement at healthcare and government facilities. Despite this, rigorous efforts are made to maximize participation among eligible women within the Study Areas.

Enrolled children's follow-ups extend until they turn 13 years old. These follow-ups primarily involve self-administered questionnaires. The JECS prioritizes early enrollment during pregnancy's early stages. Questionnaires are dispensed to enrolled mothers and partners during first and subsequent trimesters, followed by semi-annual distributions.

Developmental Assessment
J-ASQ-3 questionnaire
Communication Skills: This includes asking questions, talking clearly, making friends, and sharing personal details.
Gross Motor Skills: These involve coordination, balance, strength, knowing where your body is in space, and reacting quickly.
Fine Motor Skills: This refers to using tools like scissors, keyboards, and rulers, as well as tasks like holding a pen, drawing, and writing neatly.
Problem-Solving: This means being creative, making decisions, being reliable, and working well in a team.
Personal Skills: These include being dependable, adaptable, and motivated.
Social Skills: This covers sharing, cooperating, making eye contact, listening well, respecting personal space, and having good manners.

Potential Confounders
Information on maternal age at delivery, pre-pregnancy body mass index, infertility treatment, maternal gestational diabetes, co-medications (e.g., antibiotics, iron, and folic acid), gestational weeks, child's sex, and birth weight were retrieved from medical records. Information on marital status, maternal and paternal smoking during pregnancy, maternal alcohol during pregnancy, maternal education, annual household income, maternal psychological distress (Kessler six-item psychological distress scale [K6 scale]) during pregnancy, maternal history of pre-pregnancy asthma, child's nursery attendance at 1 year of age, and breastfeeding status were collected via self-administered questionnaires. The Japanese version of the K6 scale was used to assess depressive tendencies, and the cutoff value was set at 5 points or more.[1] A score above the cutoff value of K6 (≥5 points) indicated poor mental health.

© 2023 Nagata A et al. *JAMA Network Open*

eTable 1. Validated J-ASQ-3 Cutoff Values

J-ASQ-3 subscale					
Child age (month)	Communication	Gross motor	Fine motor	Problem solving	Personal-social
6	22.93	15.12	16.24	26.27	7.88
12	4.53	9.43	25.47	15.37	4.95
18	5.82	37.59	26.76	15.93	24.57
24	14.33	39.13	33.48	29.38	25.22
30	26.01	38.36	21.03	25.78	29.70
36	29.95	39.26	27.91	30.03	29.89

Abbreviations: J-ASQ-3, Japanese version of the Ages and Stages Questionnaires, 3rd edition

© 2023 Nagata A et al. *JAMA Network Open*

eTable 2. Association of Pre-Pregnancy Exposure To Corticosteroids and β2AAs With Offspring Neurodevelopmental Milestones

		Adjusted[a] OR (95% CI)				
		J-ASQ-3 subscale				
**Corticosteroids**	**No. (%)**	**Communication**	**Gross Motor**	**Fine Motor**	**Problem solving**	**Personal social**
Unexposed, during pregnancy	87 677 (97.9)	1 [Reference]	1 [Reference]	1 [Reference]	1 [Reference]	1 [Reference]
Exposed, pre-pregnancy[b]	1 879 (2.1)	0.94 (0.74-1.19)	0.96 (0.83-1.11)	1.01 (0.87-1.18)	1.02 (0.89-1.16)	1.02 (0.82-1.27)
**β2AAs**						
Unexposed, during pregnancy	90 278 (99.5)	1 [Reference]	1 [Reference]	1 [Reference]	1 [Reference]	1 [Reference]
Exposed, pre-pregnancy[b]	434 (0.5)	0.54 (0.28-1.01)	0.97 (0.73-1.28)	0.79 (0.59-1.04)	0.73 (0.53-1.01)	0.72 (0.42-1.22)

Abbreviations: β2AAs, β2-Adrenergic agonists; ORs, odds ratio; CI, confidence interval; and J-ASQ-3, Japanese version of the Ages and Stages Questionnaires, 3rd edition
[a] Adjusted for maternal age at delivery, marital status, educational level, history of pre-pregnancy asthma, alcohol consumption during pregnancy, maternal and paternal smoking during pregnancy, household annual income, and offspring sex.
[b] One-year prior pregnancy, constitutes as a negative control.

© 2023 Nagata A et al. *JAMA Network Open*

eTable 3. Sensitivity Analysis on the Association of In-Utero Exposure to Corticosteroids and β2AAs With Offspring Neurodevelopmental Milestones in Additionally Adjusted GEE Models

		Adjusted[a] OR (95% CI)				
		J-ASQ-3 subscale				
**Corticosteroids**	**No. (%)**	**Communication**	**Gross Motor**	**Fine Motor**	**Problem solving**	**Personal social**
Unexposed, during pregnancy	87 677 (98.0)	1 [Reference]	1 [Reference]	1 [Reference]	1 [Reference]	1 [Reference]
Exposed, early pregnancy	401 (0.4)	0.91 (0.53-1.55)	0.81 (0.57-1.13)	0.84 (0.61-1.14)	0.92 (0.67-1.25)	0.85 (0.50-1.28)
Exposed, mid- to late pregnancy	935 (1.0)	1.05 (0.74-1.48)	0.93 (0.74-1.16)	0.92 (0.74-1.15)	0.95 (0.76-1.17)	0.89 (0.63-1.25)
Exposed, both early and mid- to late pregnancy	568 (0.6)	1.05 (0.70-1.59)	1.02 (0.78-1.33)	1.10 (0.86-1.41)	1.05 (0.82-1.34)	0.80 (0.50-1.28)
**β2AAs**						
Unexposed, during pregnancy	90 278 (99.2)	1 [Reference]	1 [Reference]	1 [Reference]	1 [Reference]	1 [Reference]
Exposed, early pregnancy	170 (0.2)	0.73 (0.23-2.27)	1.05 (0.60-1.83)	0.71 (0.41-1.23)	0.87 (0.53-1.43)	0.74 (0.26-2.09)
Exposed, mid- to late pregnancy	394 (0.4)	1.46 (0.89-2.38)	1.30 (0.91-1.85)	1.28 (0.89-1.86)	1.22 (0.86-1.71)	1.59 (1.00-2.53)[b]
Exposed, both early and mid- to late pregnancy	184 (0.2)	1.17 (0.57-2.40)	0.80 (0.52-1.22)	0.77 (0.48-1.23)	0.98 (0.63-1.51)	0.49 (0.24-0.99)

Abbreviations: β2AAs, β2-Adrenergic agonists; ORs, odds ratio; CI, confidence interval; and J-ASQ-3, Japanese version of the Ages and Stages Questionnaires, 3rd edition

[a] Adjusted for maternal age at delivery, marital status, educational level, history of pre-pregnancy asthma, alcohol consumption during pregnancy, maternal and paternal smoking during pregnancy, household annual income, offspring sex, maternal pre-pregnancy body mass index, infertility treatment, psychological distress during pregnancy, gestational diabetes, comedications (antibiotics, iron, and folic acid), gestational weeks, birth weight, breastfeeding, and nursery attendance.

[b] $p < .05$

© 2023 Nagata A et al. *JAMA Network Open*

eTable 4. Sensitivity Analysis on the Association of In-Utero Exposure to Corticosteroids and β2AAs With Offspring Neurodevelopmental Milestones in GEE Models With Imputed Missing Values

	Adjusted[a] OR (95% CI)				
	J-ASQ-3 subscale				
**Corticosteroids**	**Communication**	**Gross Motor**	**Fine Motor**	**Problem solving**	**Personal social**
Unexposed, during pregnancy	1 [Reference]	1 [Reference]	1 [Reference]	1 [Reference]	1 [Reference]
Exposed, early pregnancy	0.87 (0.58-1.32)	0.82 (0.63-1.06)	0.89 (0.69-1.13)	0.97 (0.76-1.24)	0.87 (0.61-1.26)
Exposed, mid- to late pregnancy	1.07 (0.83-1.35)	0.92 (0.79-1.06)	0.97 (0.83-1.13)	0.95 (0.76-1.17)	0.87 (0.60-1.20)
Exposed, both early and mid- to late pregnancy	1.13 (0.83-1.52)	0.97 (0.79-1.20)	1.08 (0.89-1.31)	1.06 (0.88-1.27)	0.84 (0.55-1.27)
**β2AAs**					
Unexposed, during pregnancy	1 [Reference]	1 [Reference]	1 [Reference]	1 [Reference]	1 [Reference]
Exposed, early pregnancy	0.72 (0.34-1.49)	0.87 (0.59-1.29)	0.90 (0.62-1.28)	0.90 (0.54-1.25)	0.71 (0.36-1.56)
Exposed, mid- to late pregnancy	0.95 (0.64-1.40)	1.02 (0.80-1.30)	1.06 (0.82-1.37)	0.98 (0.77-1.24)	1.19 (0.98-1.43)
Exposed, both early and mid- to late pregnancy	1.04 (0.60-1.81)	0.73 (0.52-1.32)	0.69 (0.46-1.27)	0.79 (0.57-1.16)	0.46 (0.24-1.05)

Abbreviations: β2AAs, β2-Adrenergic agonists; ORs, odds ratio; CI, confidence interval; and J-ASQ-3, Japanese version of the Ages and Stages Questionnaires, 3rd edition
[a] Adjusted for maternal age at delivery, marital status, educational level, history of pre-pregnancy asthma, alcohol consumption during pregnancy, maternal and paternal smoking during pregnancy, household annual income, and offspring sex.

© 2023 Nagata A et al. *JAMA Network Open*

eTable 5. Sensitivity Analysis on the Association of In-Utero Exposure to Corticosteroids and β2AAs With Offspring Neurodevelopmental Milestones in GEE Models With Sample Mean Less Than (2×SD) as Cutoff Values for Each J-ASQ-3 Domain

	Adjusted[a] OR (95% CI)				
	J-ASQ-3 subscale				
**Corticosteroids**	**Communication**	**Gross Motor**	**Fine Motor**	**Problem solving**	**Personal social**
Unexposed, during pregnancy	1 [Reference]	1 [Reference]	1 [Reference]	1 [Reference]	1 [Reference]
Exposed, early pregnancy	1.16 (0.79-1.72)	0.79 (0.53-1.20)	0.84 (0.60-1.17)	0.96 (0.69-1.33)	0.95 (0.65-1.13)
Exposed, mid- to late pregnancy	1.05 (0.80-1.37)	0.93 (0.72-1.20)	1.03 (0.81-1.30)	0.98 (0.78-1.24)	0.86 (0.65-1.13)
Exposed, both early and mid- to late pregnancy	1.01 (0.71-1.43)	1.15 (0.86-1.55)	1.17 (0.90-1.52)	1.14 (0.85-1.51)	1.26 (0.92-1.72)
**β2AAs**					
Unexposed, during pregnancy	1 [Reference]	1 [Reference]	1 [Reference]	1 [Reference]	1 [Reference]
Exposed, early pregnancy	0.99 (0.44-2.23)	0.93 (0.51-1.72)	0.69 (0.34-1.41)	0.88 (0.49-1.58)	0.70 (0.31-1.57)
Exposed, mid- to late pregnancy	1.19 (0.76-1.86)	1.22 (0.80-1.85)	1.28 (0.88-1.85)	1.20 (0.82-1.77)	1.34 (0.89-2.01)
Exposed, both early and mid- to late pregnancy	1.06 (0.57-1.97)	0.85 (0.52-1.39)	0.79 (0.46-1.35)	0.96 (0.58-1.58)	0.93 (0.52-1.67)

Abbreviations: β2AAs, β2-Adrenergic agonists; ORs, odds ratio; CI, confidence interval; and J-ASQ-3, Japanese version of the Ages and Stages Questionnaires, 3rd edition
[a] Adjusted for maternal age at delivery, marital status, educational level, history of pre-pregnancy asthma, alcohol consumption during pregnancy, maternal and paternal smoking during pregnancy, household annual income, and offspring sex.

© 2023 Nagata A et al. *JAMA Network Open*

eTable 6. Sensitivity Analysis on the Association of In-Utero Exposure to Corticosteroids and β2AAs With offspring Neurodevelopmental Milestones in Linear Multivariable GEE Models

	**β (95% CI)**				
	**J-ASQ-3 subscale**				
**Corticosteroids**	**Communication**	**Gross Motor**	**Fine Motor**	**Problem solving**	**Personal social**
Unexposed, during pregnancy	0 [Reference]	0 [Reference]	0 [Reference]	0 [Reference]	0 [Reference]
Exposed, early pregnancy	-0.63 (-1.73; 0.47)	0.37 (-0.59; 1.33)	0.05 (-0.87; 0.97)	-0.32 (-1.33; 0.67)	-0.08 (-0.74; 0.57)
Exposed, mid- to late pregnancy	-1.04 (-1.84; 0.13)	0.26 (-0.38; 0.91)	0.08 (-0.59; 0.76)	-0.08 (-0.80; 0.62)	-0.08 (-0.74; 0.57)
Exposed, both early and mid- to late pregnancy	0.36 (-0.56; 1.29)	-0.30 (-1.19; 0.59)	-0.38 (-1.26; 0.49)	-0.08 (-1.01; 0.84)	-0.19 (-1.07; 0.68)
**β2AAs**					
Unexposed, during pregnancy	0 [Reference]	0 [Reference]	0 [Reference]	0 [Reference]	0 [Reference]
Exposed, early pregnancy	1.38 (-0.54; 3.30)	0.47 (-1.16; 2.12)	1.59 (-0.00; 3.18)	1.02 (-0.81; 2.87)	1.08 (-0.48; 2.65)
Exposed, mid- to late pregnancy	-0.91 (-2.26; 0.43)	-0.12 (-1.34; 1.09)	-0.35 (-1.59; 0.88)	-0.32 (-1.57; 0.92)	-0.30 (-1.50; 0.90)
Exposed, both early and mid- to late pregnancy	-0.82 (-2.72; 1.08)	1.03 (-0.51; 2.57)	0.54 (-1.03; 2.12)	0.44 (-1.19; 2.07)	0.35 (-1.21; 1.92)

Abbreviations: β2AAs, β2-Adrenergic agonists; ORs, odds ratio; CI, confidence interval; and J-ASQ-3, Japanese version of the Ages and Stages Questionnaires, 3rd edition
[a] Adjusted for maternal age at delivery, marital status, educational level, history of pre-pregnancy asthma, alcohol consumption during pregnancy, maternal and paternal smoking during pregnancy, household annual income, and offspring sex.

© 2023 Nagata A et al. *JAMA Network Open*

eTable 7. Subgroup Analysis of the Association of In-Utero Exposure to Corticosteroids and β2AAs With Offspring Developmental Milestones According to the Child's Sex

		Corticosteroids			β2AAs		
**Corticosteroids**		**Males**	**Females**		**Males**	**Females**	
**Timing of exposure**	J-ASQ-3 subscale	Adjusted[a] OR (95% CI)	Adjusted[a] OR (95% CI)	*P* for interaction	Adjusted[a] OR (95% CI)	Adjusted[a] OR (95% CI)	*P* for interaction
Unexposed, during pregnancy		1 [Reference]	1 [Reference]		1 [Reference]	1 [Reference]	
Exposed, early pregnancy	Communication	0.87 (0.45–1.69)	1.23 (0.50–2.05)	.32	0.11 (0.01–0.77)	2.47 (0.70–8.64)	.06
	Gross motor	0.86 (0.53–1.38)	0.79 (0.48–1.28)	.85	1.33 (0.64–2.77)	0.71 (0.30–1.68)	.42
	Fine motor	1.02 (0.71–1.45)	0.56 (0.28–1.11)	.07	0.51 (0.22–1.17)	1.00 (0.47–2.13)	.41
	Problem solving	1.08 (0.75–1.56)	0.71 (0.41–1.22)	.11	0.66 (0.34–1.28)	1.12 (0.52–2.02)	.69
	Personal-social	0.99 (0.55–1.78)	0.70 (0.30–1.61)	.31	0.45 (0.14–1.46)	1.35 (0.26–6.92)	.61
Exposed, mid- to late pregnancy	Communication	1.05 (0.70–1.58)	1.08 (0.59–1.99)	.64	1.10 (1.14–2.16)	0.79 (0.59–1.23)	.87
	Gross motor	0.98 (0.73–1.32)	0.93 (0.67–1.29)	.61	1.54 (0.95–2.50)	0.96 (0.59–1.57)	.28
	Fine motor	0.99 (0.75–1.29)	0.83 (0.56–1.29)	.10	1.66 (1.08–2.54)	0.63 (0.30–1.30)	.27
	Problem solving	0.99 (0.77–1.29)	0.90 (0.62–1.29)	.50	1.65 (1.11–2.45)	0.55 (0.30–0.99)	.00[b]
	Personal-social	0.83 (0.56–1.22)	1.12 (0.59–2.15)	.59	1.70 (0.97–2.96)	1.09 (0.53–2.23)	.53
Exposed, both early and mid- to late pregnancy	Communication	0.99 (0.58–1.68)	1.61 (0.86–3.02)	.62	0.64 (0.16–2.58)	3.03 (1.43–6.45)	.10
	Gross motor	1.11 (0.77–1.59)	1.09 (0.73–1.64)	.82	0.83 (0.47–1.49)	0.76 (0.40–1.46)	.74
	Fine motor	1.19 (0.87–1.62)	1.12 (0.74–1.68)	.56	0.80 (0.45–1.43)	0.75 (0.33–1.69)	.53
	Problem solving	1.11 (0.81–1.52)	1.16 (0.79–1.70)	.60	0.84 (0.47–1.51)	1.25 (0.52–2.40)	.37
	Personal-social	0.87 (0.51–1.51)	0.96 (0.39–2.33)	.97	0.42 (0.16–1.10)	0.78 (0.25–2.36)	.99

Abbreviations: β2AAs, β2-Adrenergic agonists; ORs, odds ratio; CI, confidence interval; and J-ASQ-3, Japanese version of the Ages and Stages Questionnaires, 3rd edition

[a] Adjusted for maternal age at delivery, marital status, educational level, history of pre-pregnancy asthma, alcohol consumption during pregnancy, maternal and paternal smoking during pregnancy, and household annual income.

[b] $p < .01$

© 2023 Nagata A et al. *JAMA Network Open*

eTable 8. Subgroup Analysis of the Association of In-Utero Exposure to Corticosteroids and β2AAs With offspring Neurodevelopmental Milestones According to the Maternal History of Pre-Pregnancy Asthma

		Corticosteroids			β2AAs		
**Corticosteroids**		**With asthma**	**Without asthma**		**With asthma**	**Without asthma**	
**Timing of exposure**	J-ASQ-3 subscale	Adjusted[a] OR (95% CI)	Adjusted[a] OR (95% CI)	*P* for interaction	Adjusted[a] OR (95% CI)	Adjusted[a] OR (95% CI)	*P* for interaction
Unexposed, during pregnancy		1 [Reference]	1 [Reference]		1 [Reference]	1 [Reference]	
Exposed, early pregnancy	Communication	1.44 (0.72–2.86)	0.63 (0.27–1.46)	.06	1.10 (0.31–3.86)	0.20 (0.03–1.45)	.03[b]
	Gross motor	0.92 (0.52–1.61)	0.81 (0.53–1.22)	.75	1.32 (0.67–2.07)	0.70 (0.25–1.98)	.23
	Fine motor	0.75 (0.45–1.26)	1.01 (0.68–1.51)	.55	0.65 (0.30–3.38)	0.84 (0.37–1.89)	.59
	Problem solving	1.06 (0.65–1.74)	0.93 (0.64–1.37)	.70	1.03 (0.56–1.91)	0.68 (0.28–1.65)	.18
	Personal-social	1.16 (0.53–2.52)	0.77 (0.43–1.39)	.93	0.82 (0.20–3.33)	0.61 (0.14–2.58)	.95
Exposed, mid- to late pregnancy	Communication	1.14 (0.68–1.91)	1.06 (0.66–1.68)	.84	1.08 (0.50–2.15)	1.62 (0.83–3.13)	.55
	Gross motor	1.15 (0.84–1.57)	0.88 (0.64–1.20)	.22	1.11 (0.70–1.75)	1.46 (0.87–2.42)	.56
	Fine motor	1.03 (0.75–1.42)	0.83 (0.56–1.29)	.29	1.01 (0.57–1.79)	1.51 (0.92–1.42)	.75
	Problem solving	1.08 (0.78–1.50)	0.96 (0.72–1.27)	.28	1.14 (0.71–1.84)	1.25 (0.77–2.04)	.87
	Personal-social	1.03 (0.66–1.59)	0.89 (0.54–1.47)	.55	1.56 (0.90–2.70)	1.47 (0.70–3.11)	.87
Exposed, both early and mid- to late pregnancy	Communication	1.32 (0.79–2.22)	1.61 (0.86–3.02)	.06	1.46 (0.69–3.09)	0.49 (0.07–3.30)	.13
	Gross motor	1.17 (0.84–1.63)	1.14 (0.71–1.83)	.86	0.99 (0.63–1.55)	0.20 (0.03–1.35)	.09
	Fine motor	1.26 (0.91–1.71)	1.17 (0.79–1.75)	.83	0.86 (0.52–1.41)	0.73 (0.17–3.02)	.93
	Problem solving	1.37 (1.01–1.84)	0.97 (0.62–1.52)	.28	1.09 (0.67–1.78)	1.01 (0.38–2.63)	.35
	Personal-social	1.11 (0.63–1.97)	0.66 (0.28–1.53)	.19	0.57 (0.26–1.25)	0.47 (0.06–3.24)	.36

Abbreviations: β2AAs, β2-Adrenergic agonists; ORs, odds ratio; CI, confidence interval; and J-ASQ-3, Japanese version of the Ages and Stages Questionnaires, 3rd edition
[a] Adjusted for maternal age at delivery, marital status, educational level, alcohol consumption during pregnancy, maternal and paternal smoking during pregnancy, household annual income and offspring sex.
[b] p <.05

© 2023 Nagata A et al. *JAMA Network Open*

eFigure 1. Directed Acyclic Graph for the Association Between In-Utero Corticosteroid and β2AA Exposure and Offspring Neurodevelopment

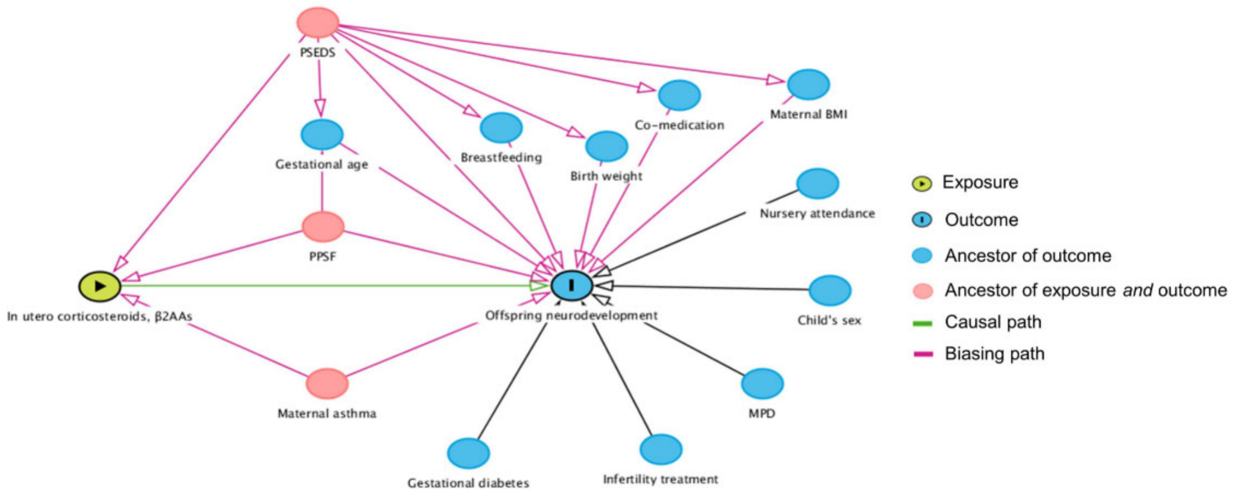

Directed acyclic graph showing the minimum sufficient set of potential confounders (red nodes) in the association between in-utero corticosteroid and β2AA exposure and offspring neurodevelopment. Blue nodes: variables associated with neurodevelopment in the offspring. Mediators are not included.
Co-medications: antibiotics, iron, and folic acid
β2AAs, β2-adrenergic agonists; PSEDS, parental socioeconomic and demographic status (maternal age, marital status, maternal educational level, household annual income); PPSF, parental psychosocial factors (maternal and paternal smoking and alcohol consumption during pregnancy); BMI, body mass index (pre-pregnancy); maternal asthma; maternal history of pre-pregnancy asthma, and MPD, maternal psychological distress during pregnancy

© 2023 Nagata A et al. *JAMA Network Open*

eFigure 2. Scores of J-ASQ-3 According to Age

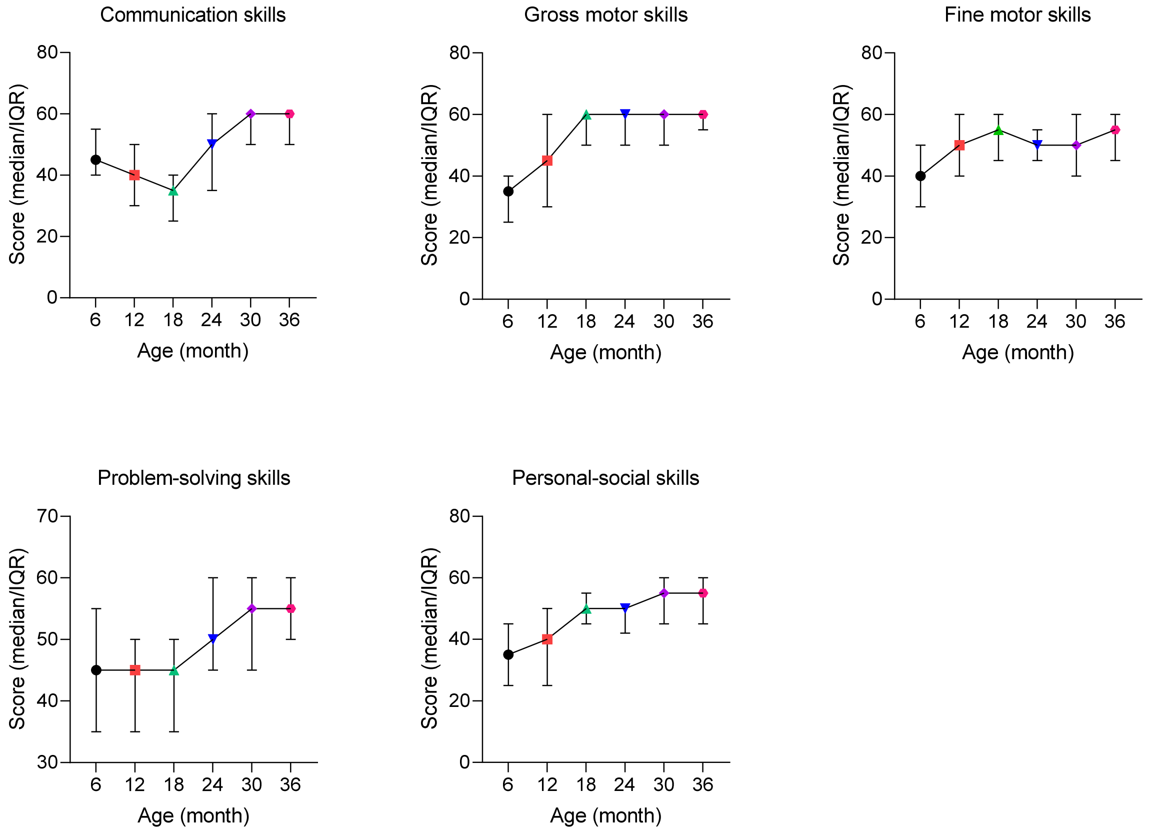

Abbreviations: J-ASQ-3, Japanese version of the Ages and Stages Questionnaires, 3rd edition; IQR, interquartile range

© 2023 Nagata A et al. *JAMA Network Open*

eReference

1. Furukawa TA, Kawakami N, Saitoh M, et al. The performance of the Japanese version of the K6 and K10 in the World Mental Health Survey Japan. *Int J Methods Psychiatr Res.* 2008;17(3):152-158.
