# Supplementary material for: Neurodevelopmental Outcomes Among Offspring Exposed to Corticosteroid and B2-Adrenergic Agonists In Utero
Source: JAMA Netw Open. 2023 Oct 24;6(10):e2339347. doi: 10.1001/jamanetworkopen.2023.39347 (PMC10599123; doi:10.1001/jamanetworkopen.2023.39347)
Supplement: Supplement 3. — Data Sharing Statement [file jamanetwopen-e2339347-s003.pdf]

Data Sharing Statement

Nagata. Association of In Utero Corticosteroid and β2-Adrenergic Agonist Exposure With Offspring Neurodevelopmental Outcomes. *JAMA Netw Open*. Published October 24, 2023. doi:10.1001/jamanetworkopen.2023.39347

Data
Data available: No

Additional Information
Explanation for why data not available: Data are unsuitable for public deposition due to ethical restrictions and legal framework of Japan. It is prohibited by the Act on the Protection of Personal Information (Act No. 57 of 30 May 2003, amendment on 9 September 2015) to publicly deposit the data containing personal information. Ethical Guidelines for Medical and Health Research Involving Human Subjects enforced by the Japan Ministry of Education, Culture, Sports, Science and Technology and the Ministry of Health, Labour and Welfare also restricts the open sharing of the epidemiologic data. All inquiries about access to data should be sent to: jecs-en@nies.go.jp. The person responsible for handling enquiries sent to this e-mail address is Dr Shoji F. Nakayama, JECS Programme Office, National Institute for Environmental Studies.
